# Hippocampal Spatial Position Evaluation on MRI for Research and Clinical Practice

**DOI:** 10.1371/journal.pone.0115174

**Published:** 2014-12-12

**Authors:** Jana Mrzílkova, Antonella Koutela, Martina Kutová, Matěj Patzelt, Ibrahim Ibrahim, Dina Al-Kayssi, Aleš Bartoš, Daniela Řípová, Pavla Čermáková, Petr Zach

**Affiliations:** 1 Institute of Anatomy, Third Faculty of Medicine, Charles University, Ruská 87, 100 00 Prague 10, Czech Republic; 2 AD Center, Prague Psychiatric Center, Ustavni 91, 181 03 Prague 8 – Bohnice, Czech Republic; 3 Charles University in Prague, Third Faculty of Medicine, Teaching Hospital Královské Vinohrady, Department of Neurology, Šrobárova 50, 100 34 Prague 10, Czech Republic; 4 Alzheimer Disease Research Center, Department of Neurobiology, Care Sciences and Society, Karolinska Institutet, 141 86 Stockholm, Sweden; 5 lnternational Clinical Research Center and St.Anne's University Hospital, Pekařská 53, 656 91 Brno, Czech Republic; 6 Department of Radiodiagnostic and Interventional Radiology, Institute for Clinical and Experimental Medicine, Vídeňská 1958/9, 140 21, Prague 4, Czech Republic; Beijing Normal University, China

## Abstract

In clinical practice as well as in many volumetric studies we use different reorientations of the brain position towards x and y axis on the magnetic resonance imaging (MRI) scans. In order to find out whether it has an overall effect on the resulting 2D data, manual hippocampal area measurements and rotation variability of the brain (in two reoriented axes) and the skull were performed in 23 Alzheimer's disease patients and 31 healthy controls. After the MRI scanning, *native brain scans* (nat) were reoriented into the two different artificial planes (*anterior commissure – posterior commissure* axis (AC-PC) and *hippocampal horizontal long axis* (hipp)). Hippocampal area and temporal horn of the lateral ventricle was measured manually using freeware Image J program. We found that 1) hippocampal area of nat images is larger compared to hipp images, area of the nat images is equal to the AC-PC images and area of the hipp images is smaller compared to AC-PC images, 2) hippocampal area together with the area of the temporal horn for nat images is larger compared to hipp images, area of the hipp images is smaller compared to the AC-PC images and area of the nat images is smaller compared to the AC-PC images. The conclusion is that the measured area of the hippocampus in the native MRI is almost the same as the area of MRI reoriented only into the AC-PC axis. Therefore, when performing 2D area studies of the hippocampus or in the clinical practice we recommend usage of not-reoriented MRI images or to reorient them into the AC-PC axis. Surprising finding was that rotation of both AC-PC and hipp line towards x-axis among patients varies up to 35° and the same is true for the skull rotation so that it is not only a matter of the brain position.

## Introduction

Visualization of the medial temporal structures, especially the hippocampus, plays an important role in the clinical evaluation of the Alzheimer's disease (AD) [1], [2]. There are numerous methods of hippocampal atrophy classification using magnetic resonance imaging (MRI) [3]. Most of them utilize frontal sections of the hippocampus. In order to evaluate hippocampal atrophy in neurological practice, we often look at the transition from the hippocampal *alveus* into the hippocampal body which we call optimal section. At this section we can observe the area of both hippocampus as well as temporal horn of the lateral ventricle in its prime (other more frontal or dorsal sections does not fully cover structure). From our experience the severity of the hippocampal atrophy can be best scored there. However, the absolute position of the brain structures commonly used for evaluation, such as anterior and posterior commissure and the hippocampus may vary for example due to cellular changes accompanying aging process [4].

### 1.1 Head and brain stabilization and possible bias during MRI scanning

During MRI scanning of the brain the patient's head is stabilized in default position without any movements. Nevertheless, the real position of the head may differ from one case to another. It may happen so due to a simple rotation of the head or neck in the sagittal plane. If we rule out head instability during MRI scanning, among other factors could be the amount of the musculature and the adipose tissue, difference in the shape of the skull (i.e. *dolichocephaly* etc.) or the presence of *lordosis/kyphosis* of the cervical/thoracic vertebras. But according to the radiologists' reports, even the most precise instructions followed by the patient's cooperation may not guarantee the same position of the head during MRI data acquisition, which may lead to a source of variability in MRI studies [5], [6]. Interestingly, previous research investigating a similar problem in the functional magnetic resonance imaging (fMRI) studies found that head-repositioning did not decrease the reproducibility of the results [7]. We were interested in finding out whether the position of the head plays an important role in manual area measurements and volumetric software processing. When considering other brain MRI studies focused on 2D or 3D analysis it is not clear whether researchers have taken repositioning into account or not. In some articles, the authors reoriented native MRI scans into the standard orientation relative to *anterior* and *posterior commissure* line (AC-PC) [8]–[11]. In another study [12] the MRI images were adapted from a midsagittal sections to the brainstem axis. However, in several other articles it is not specified whether the head was reoriented into the midsagittal axis or not [13], [14]. In order to evaluate the influence of the head rotation in the sagittal plane on the hippocampal area measurement, we introduced one more axis – the hippocampal long axis. We compared it with the commonly used AC-PC axis and native scan (images w/o any reorientations – as they are after MRI scanning).

We aimed to investigate how much the view of the hippocampus differs in native and optimal section compared to various axial reorientations that are commonly used in research and clinical practice. We were also interested in finding out whether the position of the head can limit the reliability of the MRI evaluations. Therefore, our goals were firstly to evaluate the rotation of the head in sagittal axis by comparing hippocampal area measurement of native images to the standardized AC-PC axis reoriented images and to the hippocampal long axis reoriented images. Secondly, to evaluate our hypothesis that area measurements in the hippocampal long axis reorientation of MRI images would yield similar results compared to the native images and be thus more useful for clinicians, in comparison with the AC-PC axis reorientation. Thirdly, to find out how much variability during MRI scanning is there in the skull rotation.

## Material and Methods

### 2.1 Study population

This study utilized 23 patients (average age 76±6 years, 8 males and 15 females) with confirmed AD diagnosis based on the NINCDS-ADRDA criteria [15]. All patients with AD and 31 healthy seniors underwent MRI of the brain. Both groups were tested with the following neuropsychological tests: Mini-Mental State Examination (MMSE), Mattis Dementia Rating Scale, Trail Making Test version A and B, Disability Assessment in Dementia, 7-Minute Screen, verbal fluency tests and Edinburgh Handedness Inventory. According to the revised version of research criteria for the diagnosis of AD [16], we added medial temporal lobe atrophy score [12], separately for the left and right hemisphere. The control group consisted of 31 cognitively normal elderly persons (average age 82±8 years, 7 males and 24 females), who were either recruited from the Third Age University of the Charles University at Prague, Czech Republic (educational courses for seniors) or among healthy volunteers visiting the AD Center at Prague. All of them reached 55 years of age, had Czech as their native language and no self-reported memory impairments. Exclusion criteria included the history of psychiatric treatment, the use of psychoactive medications (e.g. antidepressants, neuroleptics, anxiolytics, or hypnotics), the history of unconsciousness lasting longer than 5 min, seizures, any serious brain damage (stroke, trauma, neuro-infection, operation, tumor), and drug/alcohol abuse. Normal cognitive functions were determined using the MMSE, the 7-Minute Screen and verbal fluency tests (1-min version: 3 phonemic, with the initial letters of NKP, and 3 semantic, using fruits, animal, and shopping items). For both groups, the following data were collected: detailed anamnesis, mapping potential comorbidities (hypertension, diabetes, cardiovascular diseases, hyperlipidemia, smoking, kidney and liver diseases, psychiatric illnesses, ictus, epilepsy and neurological diseases), medical treatment (antidepressants, antipsychotics, anxiolytics, hypnotics, nootropics, cognitive treatment and others) and basic demographic data (living standards, years of education, highest education). Patients with AD were followed for several years and the healthy seniors underwent neuropsychological testing bi-annually. All participants signed an informed consent. The research was approved by the Ethics Committee of the University Hospital Kralovske Vinohrady, Prague, Czech Republic.

### 2.2 MRI specifications

Three-dimensional MRI images were acquired on the scanner Siemens Trio 3 T, TQ-engine gradient system, and 18 RF channels. Acquisition parameters for the volume analysis were: 192 sagittal layers, 3D sequence MP-RAGE, resolution 0,85×0,85×0,85 mm, (FOV 326 mm, matrix 384×384), TE 4,73 ms, TR 2000 ms, TI 800 ms, declination angle 10°, bandwidth 130 Hz/pixel, acquisition time 10∶50 min.

### 2.3 Area measurement and computer analysis

**Figure 1:**
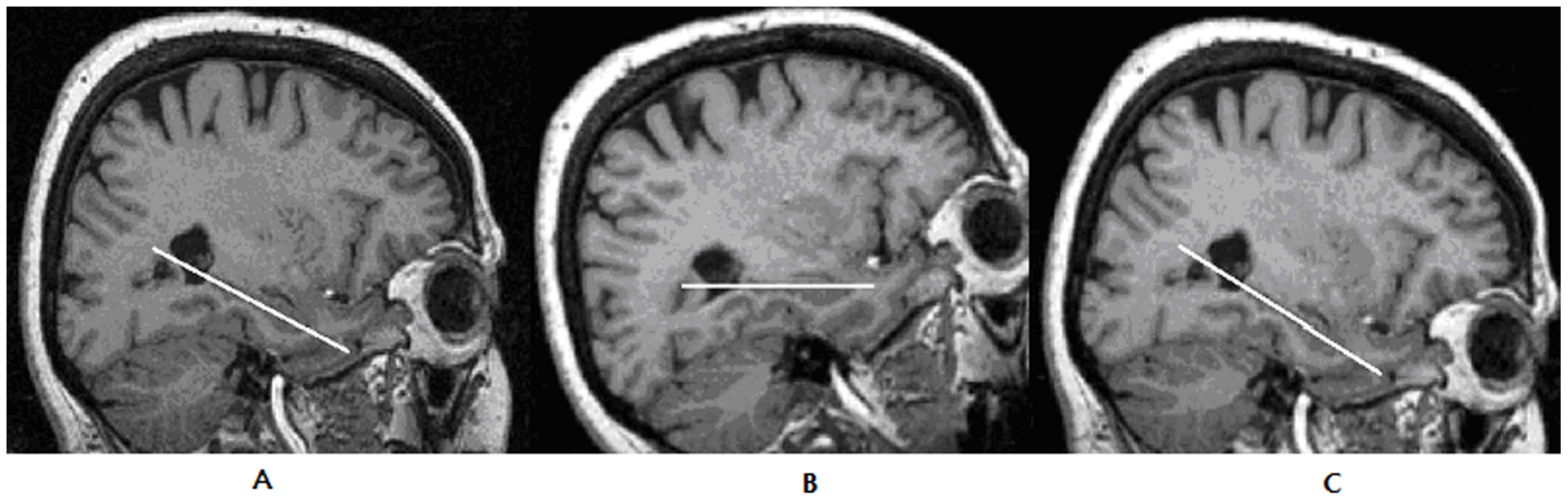
a,b,c Examples of the sagittal view of the right hippocampus on the MRI in one native and two reoriented axis. MRI sections were selected according to the best visibility of the of the hippocampus on the sagittal sections (its long or dorsoventral axis), a) position of the hippocampus on the “native” MRI scan, b) reorientation of the “native” MRI scan into the hipo-axis axis (where long axis of the hippocampus is parallel to the horizontal axis), c) reorientation of the “native” MRI scan into the CA-CP. White lines represent long axis of the hippocampus.

The MRI images were exported as a multiple data format files into a standard computer. The MRI images of the brains were then converted into the stack of files by the MRIcro freeware and analyzed on Image J freeware. The areas of the hippocampi in (mm^2^) and hippocampus with temporal horn of the lateral ventricle (both at the level of the transition of *alveus* into the hippocampal body) were manually delineated independently by two experienced neuroanatomists. Hippocampal areas solely and collectively with the temporal horn of the lateral ventricle areas were measured in the three different rotational scan stacks of the MRI. Firstly, we measured areas in the “native” (nat) unaltered MRI scan stacks (Fig. 1a) as we obtained it from the MRI scanner. Secondly, we measured areas in the MRI scan stacks that were rotated using the MRIcro program so that the hippocampal long axis in the sagittal projection was parallel to the x-axis (hipp). Finally, the MRI scan stacks once again were rotated using the MRIcro program so that the sagittal projection line connecting the AC-PC was parallel to the x – axis (AC-PC). The areas of the hippocampi and hippocampus together with temporal horn of the lateral ventricle were calculated separately and adjusted to the areas of the brain and skull at the frontal cross-section at the level of *anterior commissure*; to exclude the impact of the brain and the skull size. The areas were adjusted according to formula (area of the hippocampus/area of the brain (skull) * 100. We did not observe statistically significant differences between adjusted and non-adjusted areas (unpublished results) so that we used in the calculations data w/o brain or skull adjustment.

### 2.4 Anatomical delineation of the hippocampus and temporal horn of the lateral ventricle

**Figure 2:**
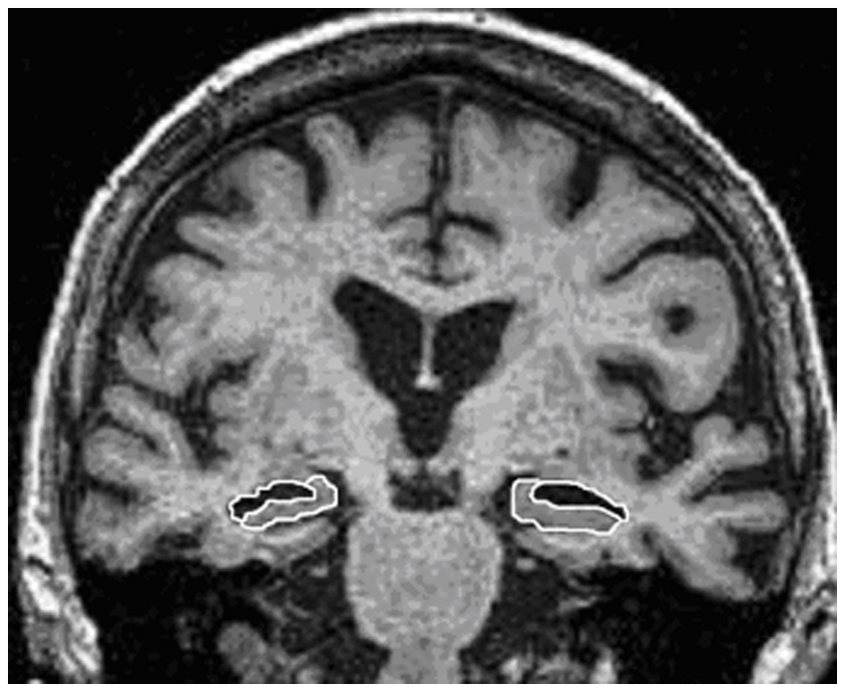
Example of manual anatomical delineation of the hippocampus (white line with grey area inside) and temporal horn of the lateral ventricle (white line with black area inside) on the left and right side. Coronal sections of the MRI are located at the transition from the *alveus* of the hippocampus into the hippocampal body.

We measured only the area of the hippocampus proper – without *subiculum* and *parahippocampal gyrus*. However, these measurements did include *dentate gyrus* due to its close position to the hippocampus on the frontal section (Fig. 2). This method is in line with a large amount of studies that use a similar protocol for hipppocampal delineation, for example [17]. The inferior border of the measured area was a clearly visible line between hippocampus and the grey matter of parahippocampal gyrus medially and directly caudally white matter of the *subiculum*. The caudo-medial border was set as cerebrospinal fluid (CSF) filled space between hippocampus and crura cerebri on both sides and cranio-medial border as the point where fornix (included in the measurement) inserts on the roof of the temporal horn of the lateral ventricle via *stria medullaris*. The lateral border of the measured area was a clearly visible round shape of the hippocampus with dark black color of the cerebrospinal fluid in the temporal horn of the lateral ventricle.

### 2.5 Rotation of the AC-PC and hipp axes of the brain

In case of AC-PC axis we measured together controls and AD patients (w/o left or right side dichotomy since AC-PC axis is midline structure without laterality) because there are no data in the literature about its impairment in the AD compared to healthy controls. On the other hand the measurement of the hipp axis rotation was performed separately in controls and AD patients (with left and right side dichotomy) due to its well known atrophy in AD compared to the controls. We opened brain scan files in MRIcro program in mid - sagittal projection and we rotated the brain so that AC-PC line was parallel to the x – axis (Fig. 1c). As next step we scrolled from the mid – sagittal projection laterally till we reached long axis of the hippocampus (Fig. 1b). At this point we got the rotation angle between long axis of the hippocampus and AC-PC line previously set parallel to x axis. This was done separately for the right and left sides.

### 2.6 Rotation of the skull (Frankfurt auriculo-orbital plane)

We evaluated the anterior-posterior rotation of the head by Frankfurt plane [18] which is the most reliable anthropological measure defining position of the human skull. We measured the angle between a line connecting the upper margin of *meatus acusticus externus* with the inferior margin of the orbita on the maxilla (at the level of equator of the eyeball) and x-axis in the MRIcro program.

## Statistics

### 3.1 Hippocampal area analysis

Statistical analysis was performed for all variables (nat, hipp and AC-PC) by the non-parametrical Friedman Analysis of Variance (ANOVA) and then each two variables together by parametrical paired t-test. In case of statistical significance we continued with Wilcoxon paired test for each grouping (hipp vs nat, AC-PC vs nat and AC-PC vs hipp). For the evaluation of the Friedman's test we did normalization by Kendall's coefficient of concordance W (0 – no agreement to 1 – complete agreement).

### 3.2 Relationship between nat, hipp and AC-PC brain rotations

**Figure 3:**
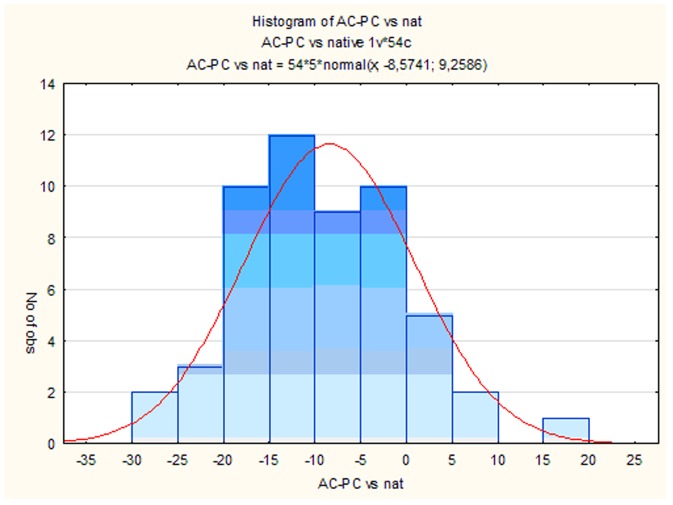
Brain rotation variability of AC-PC vs nat on MRI. The extent of the brain rotation in AC-PC vs nat is shown in decimal degrees on x-axis (– counter clockwise and + clockwise rotation) (p<0.05).

**Figure 4:**
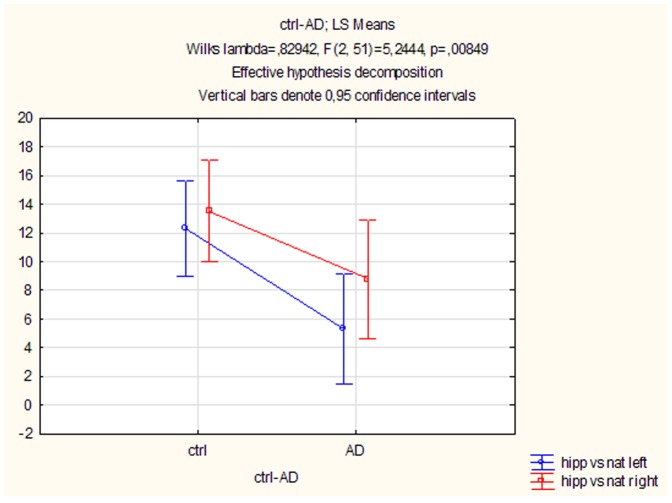
Brain rotation variability of hipp vs nat on MRI. Data showing difference between left and right hipp axes (in the left and right hemisphere). There is a significant difference between left hipp axis rotation in the AD patients compared to the controls (p = 0.008). The right hipp axis rotation in the AD patients compared to the controls are not significant (p = 0.08). ANOVA F (2, 51)  = 2.54.

Rotation of AC-PC axis vs nat axis was evaluated by the histogram (Fig. 3). Rotations of the left and right hipp axes vs nat axis (separately for controls and AD patients groups) (Fig. 4) and rotations of the AC-PC axis vs hipp axis was evaluated by parametrical paired t-test.

### 3.3 Relationship between skull rotation and AC-PC brain rotation

**Figure 5:**
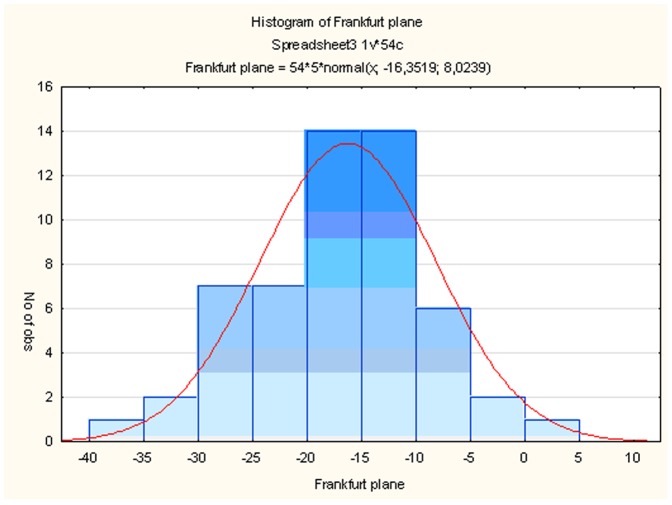
Skull rotation variability in MRI. Skull dorsal and ventral skull rotation expressed as Frankfurt plane angles in decimal degree on x-axis (- counter clockwise rotation or dorsal and + clockwise rotation or ventral).

**Figure 6:**
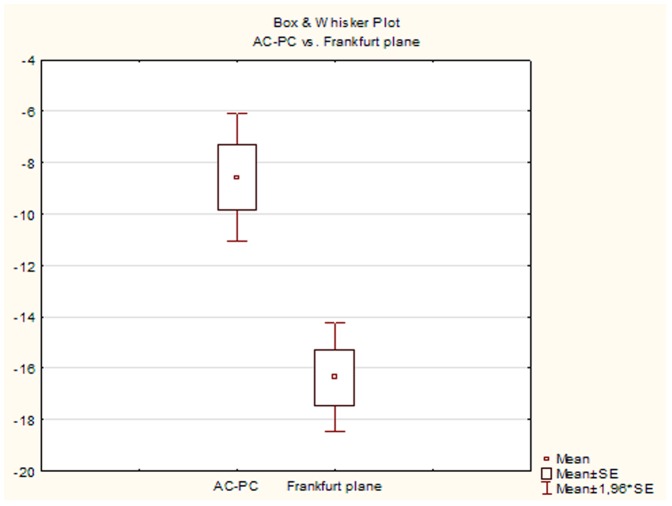
Relationship between brain and skull rotation. The brain rotation in AC-PC axis and the skull rotation (Frankfurt plane) exhibit a similar degree of variability (- counter clockwise or dorsal). AC-PC axis, Mean  = -8.57°, SE = 9.2°. Frankfurt (auriculo-orbital) plane, Mean  = −16.3°, SE = 8.02°.

The Frankfurt plane variability for the whole group was evaluated by the histogram (Fig. 5). Similarity as in the case of skull and brain rotation (for the whole group) statistics was evaluated by dependent paired t-test (Frankfurt plane vs AC-PC axis) (Fig. 6).

## Results

The area of the hippocampal cross-section was measured in 54 subjects (31 controls and 23 AD patients of heterogeneous age category) in three types of brain rotation (nat, hipp and AC-PC) so that for each brain rotation (3x) we accounted 216 area measurements (2×108 - left and right together). Hipp axis rotation variability was measured separately on the left and on the right in controls (n = 31) and AD patients (n = 23). Nat and AC-PC axes rotation variability were calculated as one group (w/o sidedness and grouping effects).

### 4.1 Effect of brain rotations on the extent of hippocampal cross section area

**Figure 7:**
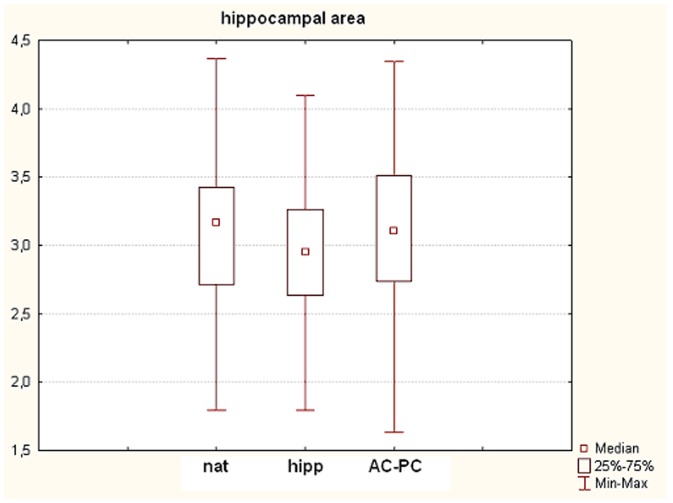
Effects of the brains rotations on the area measurements of the hippocampus on the MRI. Three groups are presented on the x-axis – nat (as the unaltered MRI scans were exported for 2D area analysis), hipp (x-axis is parallel to the long axis of the hippocampus) and AC-PC (x axis is parallel to the line connecting *anterior* and *posterior commissure*). On the y-axis, 2D area values of the hippocampi in mm^2^ are presented. Results are presented as median ± min-max.

There was no significant difference between the measured area of hippocampus in nat compared to AC-PC (p = 0.82, W = 0.17). On the other hand, the area of the hippocampus in nat was larger than the area in hipp (p<0.05, W = 0.17). The area of the hippocampus in hipp was smaller compared to the area in AC-PC (p<0.05, W = 0.17) (Fig. 7).

### 4.2 Effect of brain rotation variability on the extent of the area of hippocampus and ventricle

**Figure 8:**
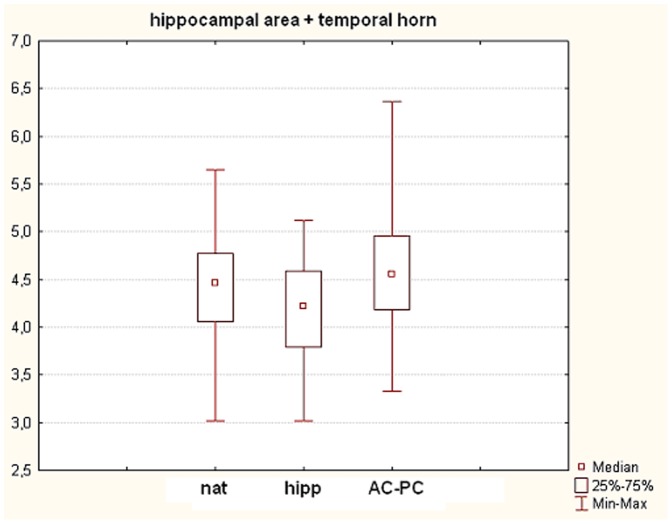
Effects of the brain rotation variability on the extent of the cross-section area of hippocampus and ventricle on the MRI. On the x-axis, three groups are presented – nat (as the unaltered MRI scans were exported for the 2D area analysis), hipp (x-axis is parallel to the long axis of the hippocampus) and AC-PC (x-axis is parallel to the line connecting *anterior* and *posterior commissure*). On the y-axis, 2D area values of the hippocampi in mm^2^ are presented. Results are presented as median ± min-max.

We found a significant difference between nat compared to hipp (p<0.05, W = 0.27) as well as between hipp compared to AC-PC (p<0.05, W = 0.27). This is on the contrary to the hippocampal area measurement only, where we found also a significant difference between nat compared to AC-PC (p<0.05, W = 0.27) (Fig. 8).

### 4.3 Relationship between nat, hipp and AC-PC brain rotations and skull rotation

The degree of the AC-PC axis rotation vs nat varies from −27° to +16° (Fig. 3). On the contrary we found that both the left and right hipp axes are rotated largely ventrally (+) and fewer dorsally (−). We also found that the left hipp axis in controls is rotated significantly more ventrally than the left hipp axis in AD patients (p = 0.008). Similarly, although without statistical significance, the right hipp axes in controls compared to AD group are also rotated more ventrally (Fig. 4).

We found that skull was rotated almost exclusively dorsally (−), only in one case ventrally (+) (Fig. 5). Comparison of the brain rotation in AC-PC axis vs skull rotation showed similar degree of variability (AC-PC, Mean −8.57°, SE 9.2° and Frankfurt plane, Mean −16.3°, SE 8.02°) (Fig. 6).

## Discussion

We evaluated effects of the rotation of the head in the sagittal plane on the manual 2D area measurements of the hippocampus that could lead to incongruent results between MRI evaluations where the manual delineation of the structures is deployed. After all we did not find any significant statistical differences in the areas of the hippocampus (AC-PC vs nat), measured on the regular coronal plane images (in the transition of the *alveus* into the body of the hippocampus). This contrasts with a simple observations made by the naked eye, where the shape of the hippocampus and temporal horn of the lateral ventricle seems different in all the three brain rotations. Besides this, neither the hippocampus nor temporal horn of lateral ventricle is rotationally symmetrical in all the geometrical projections (frontal, sagittal, horizontal). Also, we found distinct, yet not significant differences between the areas of the hippocampus compared to the hippocampus with temporal horn of lateral ventricle. This could be possibly explained by effect of the higher total area; leading to the higher impact on the overall brain position. Furthermore, we included into the measurements together the controls, AD patients, different age categories and left-right hemispheres since our interest was in the effect of the regional geometry on the areas (which is often the case in most of MRI volumetric studies). Taken these differences in categorization into account, AD patients have generally larger volumes of the temporal horn of the lateral ventricle compared to healthy controls [12]; therefore more patients with this diagnosis could have larger areas and thus possibly leading to the different results. We did not try to measure volumes of the whole hippocampus and/or whole volume of the temporal horn of lateral ventricle, since the effect of the brain rotations would manifest in terms of absolute values and this way it would be eventually nullified. Assessing our eventual experimental limitations, we worked with magnetic resonance images from the same institute and the same magnetic resonance scanner. This could add to the causes biases, since different setups and protocols in different radiology/MRI departments may lead to incongruent results. Moreover, we did not measure the effects of the lateral head/brain rotation, whose effect may have similar impact on the area measurement as the sagittal rotation.

If we stop thinking of the previously discussed rotations of the head or brain during MRI processing, further rotations could be conceived for different good reasons (i.e. change of the angle of the coronal plane of the MRI stack, to see better borders of the hippocampus, etc.) within the stereological software itself. We are not certain, whether it is possible to calculate how much we can afford to rotate the brain within the stereological software, while not changing 2D area results significantly. In another words, it is still unknown how large is the interval within which we can afford to manipulate the rotation of the brain, while still getting undistorted area results. Once this information is known, then we can rely more on inter reliability of different studies on the volumes of hippocampus, regardless of different view angles. This would be another interesting topic to be further studied. In addition to the previously discussed biases, our research was performed using only one MRI scanner, and it would be impossible to conclude that similar results could be obtained by the MRI scanners worldwide. Therefore it is necessary to perform similar measurements on different MRI scanners to find out whether these differences exist.

Unexpected finding was high inter – individual variability in the brain and skull rotations in the MRI (up to 35°). Both AC-PC vs nat as well as hipp vs nat and skull rotations showed similar trend. This finding was very surprising. In case of the skull rotation we used reliable method for its evaluation (Frankfurt plane is considered to be the most reliable skull anthropological measure) so that it would be easy to explain this phenomenon by a wrong head fixation protocol during the MRI processing. But our results from hipp vs nat rotation showed significant decrease in the dorsal brain rotation (or just its temporal lobe) in AD patients, especially on the left side. This finding is consistent with the overall hippocampal volume decrease in AD but it is showing more. It appears that hippocampal volume shrinkage may have specific effect on the position of the brain inside the skull, especially in the *middle cerebral fossa* where the temporal lobe resides. It is a matter of dispute what exactly accounts for the variability of the brain position inside the skull or if this could be attributed to the differences in the skull position or also to geometrically specific tissue shrinkage. If this is the case then any normalization of the brain positions are questionable because of the great variability in the tissue torsion, elongation and eventually not yet recognized patterns of the brain internal geometry variations. We did not find any suitable reference in the current literature describing similar anatomical features.

## Conclusions

In order to quantify the effect of 3 different brain positions (native position and position according to hippocampal long axis or AC-PC axis) on the hippocampal area measurement, we rotated the brain manually in the MRIcro program prior to the area measurements of the hippocampus alone and hippocampus with the temporal horn of the lateral ventricle. We found differences of the brain/head position in the sagittal plane in the native MRI stacks (unavoidable rotations of the head during MRI data acquisition). Also we found significant effect of the 1st artificial rotation (hipp axis) on the manual volumetric area measurements of the hippocampus and significant effect of the 2nd artificial rotation (hipp vs nat and AC-PC vs nat) of the hippocampus with temporal horn of the lateral ventricle in the frontal plane. In other words, reorientation of native MRI images into AC-PC axis does not make any significant difference on the measurements of the hippocampal area. However, this does not say other MRI scanners would produce the same results. We recommend performing similar measurements on several different MRI scanners for comparison. Regardless of this we presume, that inevitable head rotation of the patient (due to for example the size of the posterior neck muscles) does not have significant impact on the measurements of areas of the medial temporal structures. For the routine area measurements we can use “native” images and the reorientation of images into the standardized AC-PC axis is not crucial in the research or clinical practice. The head may be flexed dorsally or ventrally due to the volume of the neck muscles or adipous tissue on the back or due to different shape of the skull (dolichocephaly, microcephaly, etc.) and maybe for plenty other reasons. The brain may suffer inconsitent atrophy of the temporal lobe or the frontal lobe or cerebellum (although in the cerebellum we did not observe significant atrophical changes [2]. Also the amount and volume of the CSF in the interventricul or subarachnoid space may contribute to change in the shape or volume. Furthermore, insertion of the dura mater on the periost inside the skull may differ significantly and this may affect the volume of the subdural or epidural space. This may cause for example ventral or dorsal torsion of the frontal lobe (sometimes reffered to as Yakovlevian torsion), which would lead to the higher AC-PC rotation angle compared to the hipp rotation angle or viceversa. In the case of the temporal lobe atrophy there would be increased rotation angle between AC-PC vs hippo due to the descensus of the whole temporal lobe together with hippocampus into the *middle cerebral fossa*. Taken together we came to the conclusion, that it is hardly possible to account for all possible effects and so it is questionable if any normalizations and brain subparts delineations in volumetric studies are valid as presented in the literature. Nevertheless, we found that the area of the hippocampus in the optimal section in native is statistically comparable to the area of the hippocampus reoriented into the AC-PC, which is according to literature taken as standard procedure. For clinicians this reorientation is a relatively complicated and time consuming process, therefore we see the advantage of our results for simplification of the clinical practice.

## Supporting Information

S1 Data **Table of raw data used in the calculations.**
(XLS)
